# Supplementary material for: Multi-layered molecular profiling informs the diagnosis and targeted therapy of desmoplastic small round cell tumor
Source: Nat Commun. 2026 Apr 9;17:3397. doi: 10.1038/s41467-026-71636-0 (PMC13066396; doi:10.1038/s41467-026-71636-0)
Supplement: Supplementary file 1 — Supplementary Information [file 41467_2026_71636_MOESM1_ESM.pdf]

Supplementary Information

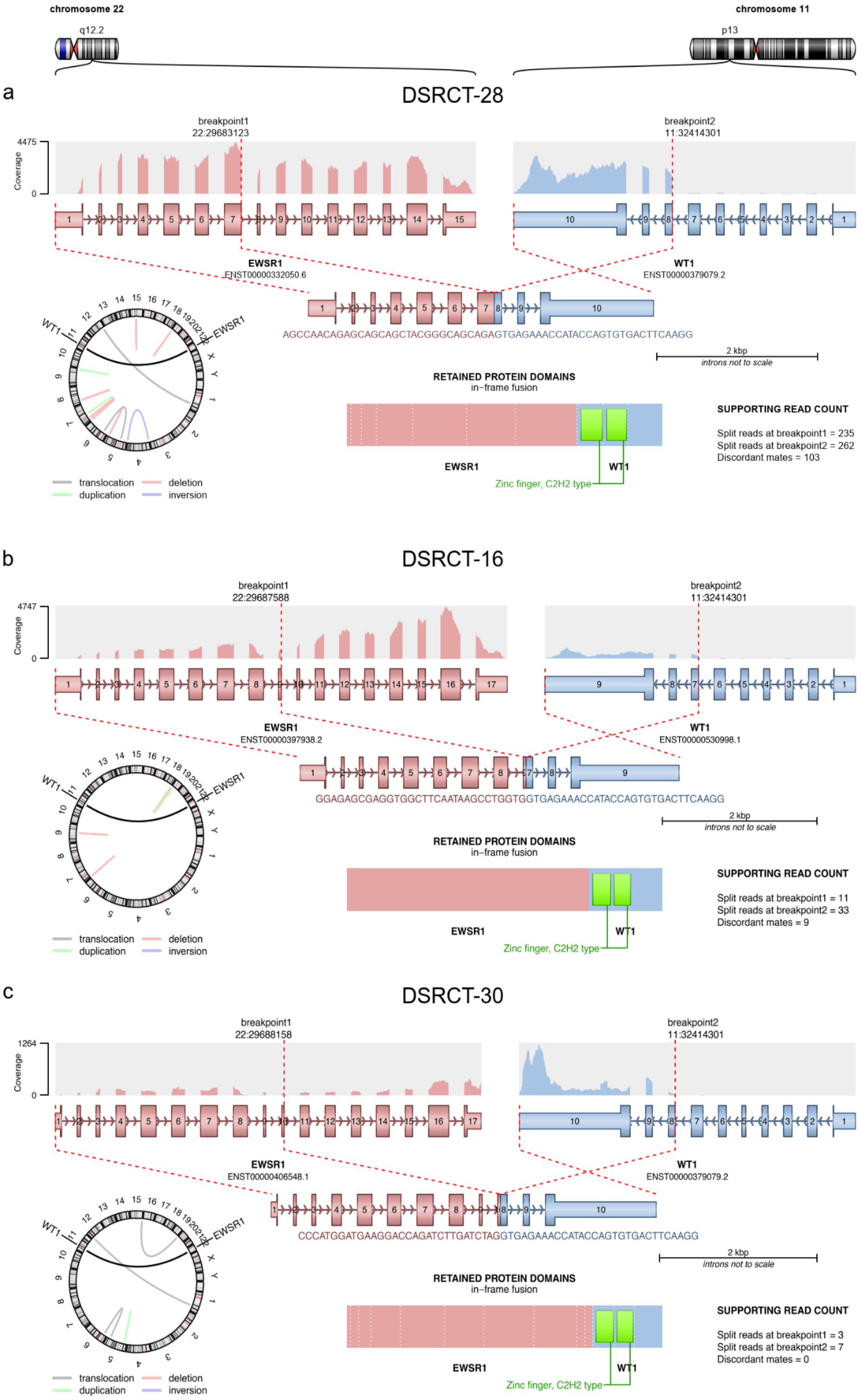

Supplementary Figure 1. Schematic representation of the three *EWSR1::WT1* fusion transcripts. **(a)** In 25 of 27 cases (92.6%), the most common *EWSR1::WT1* fusion with breakpoints in exons 7-8 was detected. Patient DSRCT-28 is shown as an example. **(b)** Patient DSRCT-16 had an *EWSR1::WT1* fusion with breakpoints in exons 9-8. **(c)** Patient DSRCT-30 had an *EWSR1::WT1* fusion with breakpoints in exons 10-8. Exon numbers are provided for the canonical *EWSR1* (ENST00000397938.7) and *WT1* (ENST00000452863.10) transcripts.

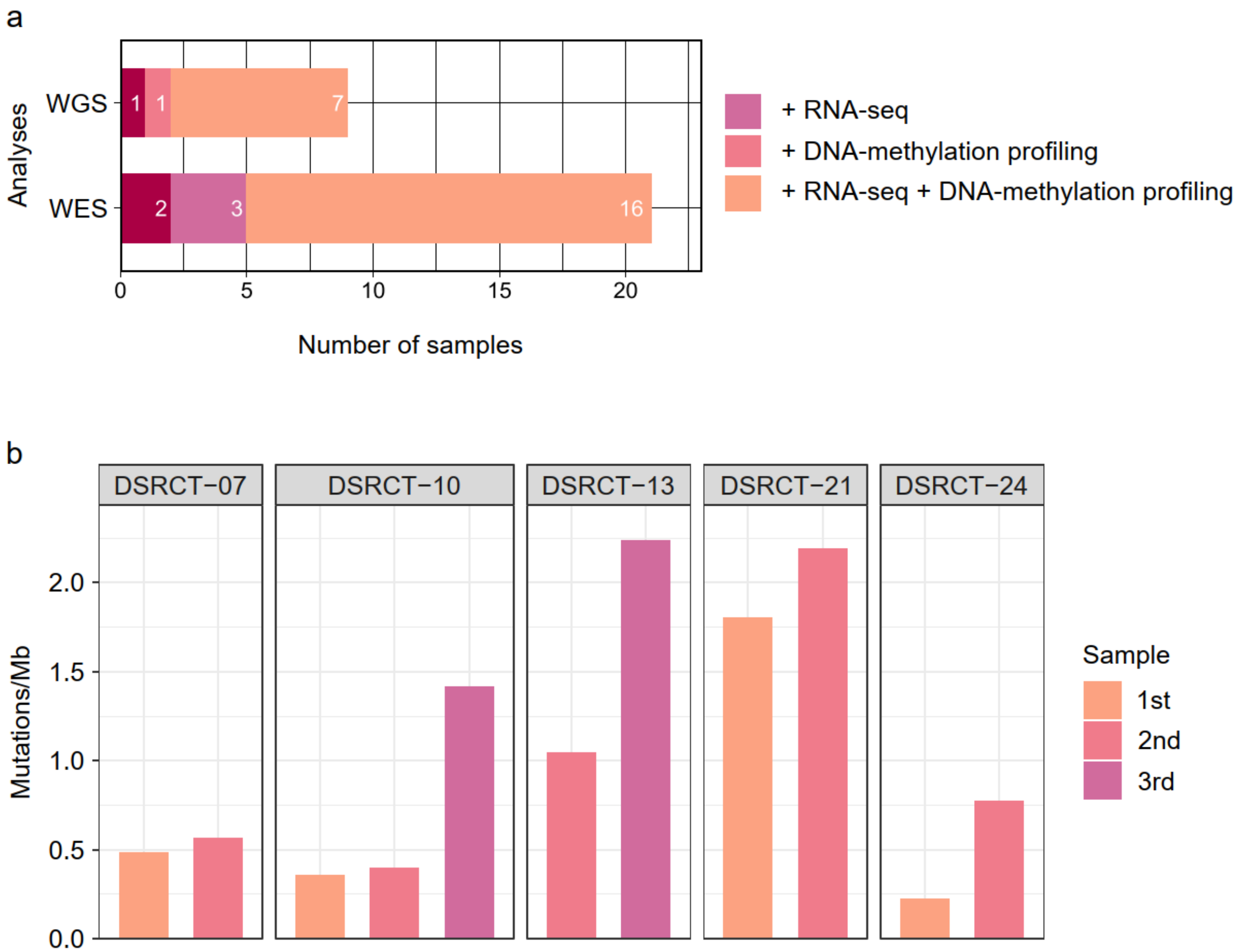

Supplementary Figure 2. (a) Number of samples analyzed by whole-exome sequencing (WES), whole-genome sequencing (WGS), RNA sequencing (RNA-seq), and DNA methylation profiling. **(b)** Increasing somatic mutation rates in five patients with sequential tumor samples. For patient DSRCT-13, the first sequenced sample was excluded due to low tumor cell content. For patient DSRCT-21, two samples are shown, although only one MTB was held, as the patient died before a second could take place. Mb, megabase. Source data for **(b)** are provided in the Source Data file.

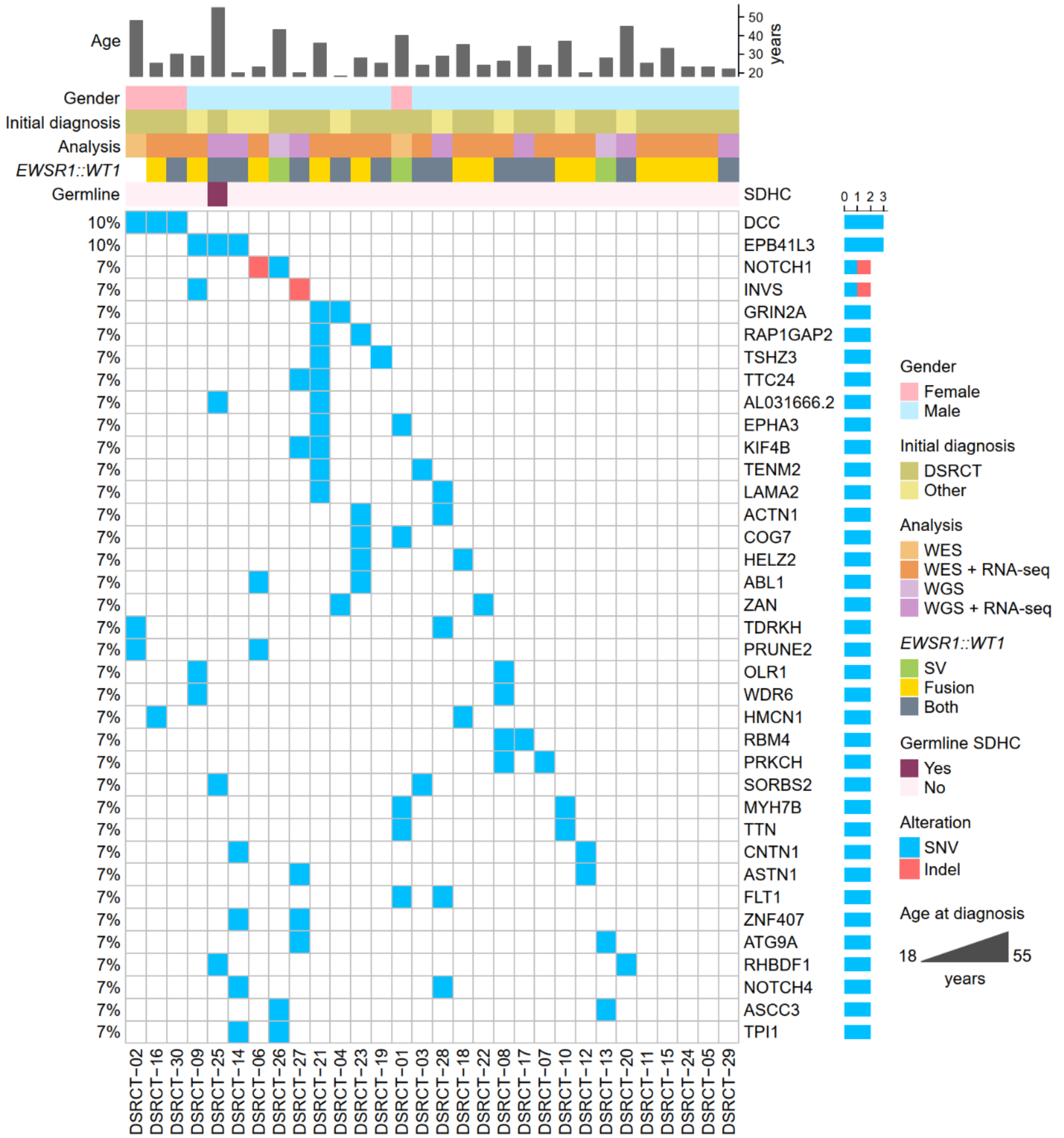

Supplementary Figure 3. Landscape of recurrent somatic SNVs and indels. The *EWSR1::WT1* fusion in patient DSRCT-02 was detected by fluorescence *in situ* hybridization in nearly all tumor cells. SV, structural variant.

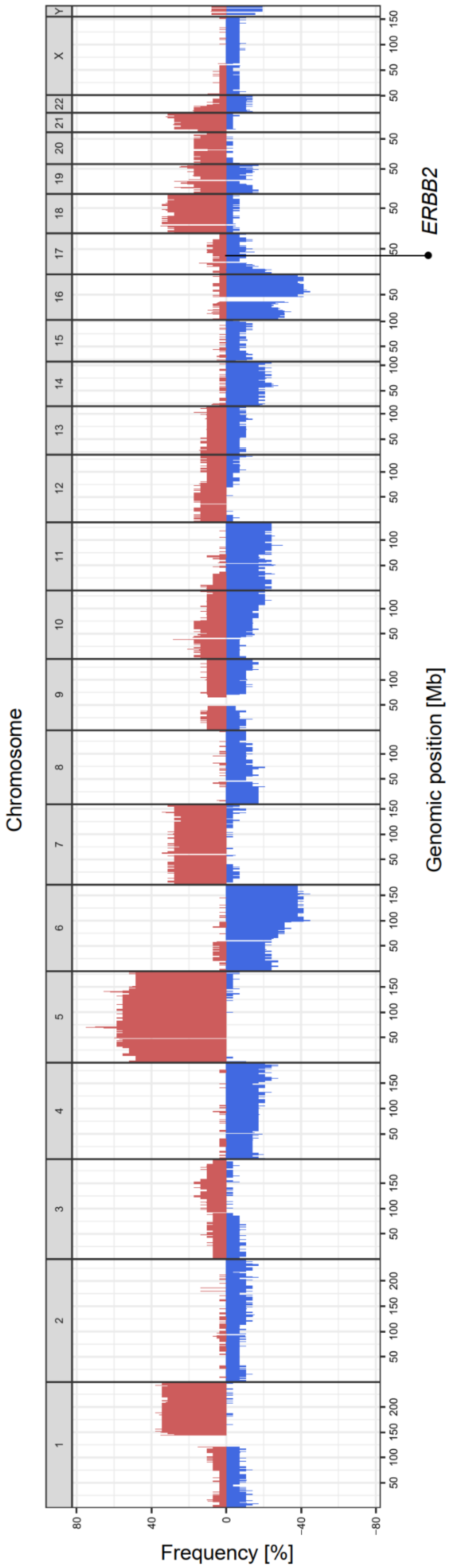

Supplementary Figure 4. Average somatic DNA copy number profile across 29 DSRCT cases. Chromosomes are represented along the vertical axis, and frequencies of genomic gains (red) and losses (blue) are represented along the horizontal axis. The position of the *ERBB2* gene on chromosome 17q12 is indicated. Mb, megabase.

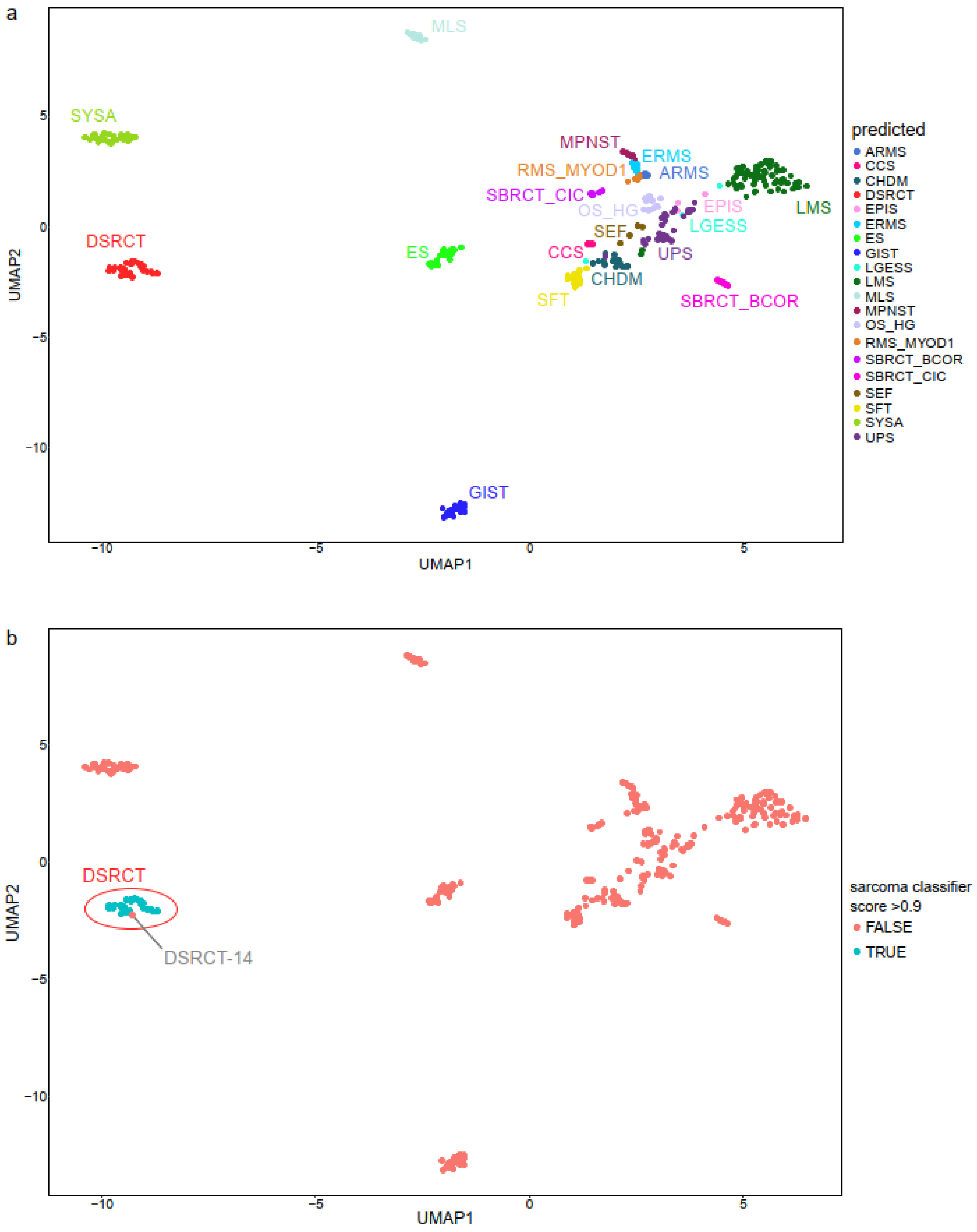

Supplementary Figure 5. (a) UMAP analysis using the 5,000 most variable CpG sites of the DNA methylation profiles of 30 DSRCT samples from 25 patients and other sarcoma samples (n = 305). Inclusion criteria for non-DSRCT samples were a sarcoma classifier score ≥ 0.9 and more than three samples per entity. **(b)** Same UMAP as in (a) with coloring according to whether the sarcoma classifier score for DSRCT was ≥ 0.9 (green) or < 0.9 (red). ARMS, alveolar rhabdomyosarcoma; CCS, clear cell sarcoma; CHDM, chordoma; DSRCT, desmoplastic small round cell tumor; EPIS, epithelioid sarcoma; LGESS, low-grade endometrial stromal sarcoma; ES, Ewing sarcoma; ERMS, embryonal rhabdomyosarcoma; GIST, gastrointestinal stromal tumor; LMS, leiomyosarcoma; MLS, myxoid liposarcoma; MPNST, malignant peripheral nerve sheath tumor; OS_HG, high-grade osteosarcoma; RMS_MYOD1, MYOD1-mutant spindle cell/sclerosing rhabdomyosarcoma; SBRCT_BCOR, small blue round cell tumor with BCOR alteration; SBRCT_CIC, small blue round cell tumor with CIC alteration; SEF, sclerosing epithelioid sarcoma; SFT, solitary fibrous tumor; SYSA, synovial sarcoma; UPS, undifferentiated pleomorphic sarcoma.

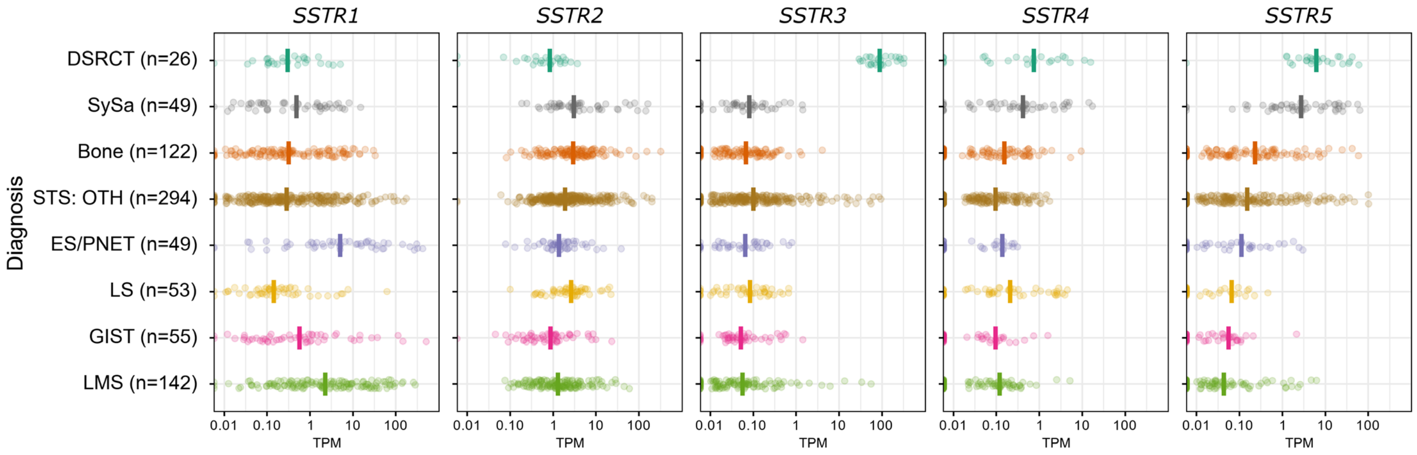

Supplementary Figure 6. *SSTR1*, *SSTR2*, *SSTR3*, *SSTR4*, and *SSTR5* mRNA expression in DSRCT compared to other sarcomas enrolled in MASTER. The vertical lines indicate median expression. GIST, gastrointestinal stromal tumor; LMS, leiomyosarcoma; LS, liposarcoma; PNET, primitive neuroectodermal tumor; SYSA, synovial sarcoma; STS, soft-tissue sarcoma; TPM, transcripts per kilobase million. Source data are provided in the Source Data file.

Supplementary Table 1. Recommendations according to intervention baskets

**Basket**	**n**	**%**
Tyrosine kinase	48	44.9
DNA damage repair	13	12.1
Immunotherapy	12	11.2
Other	12	11.2
Theranostics	11	10.3
PI3K-AKT-mTOR	6	5.6
Antibody-drug conjugate	2	1.9
Cell cycle	2	1.9
RAF-MEK-ERK	1	0.9

Supplementary Table 2. Clinical trials recommended based on molecular profiling

Patient	Molecular tumor board	ClinicalTrials.gov identifier(s)
DSRCT-07	2	NCT02987959, NCT02576444
DSRCT-12	1	NCT02568267
DSRCT-13	3	NCT03127215, NCT04503278
DSRCT-15	1	NCT01976741
DSRCT-16	1	NCT01976741, NCT01189643, NCT02460224, NCT01968109
DSRCT-17	1	NCT03127215, NCT02419417
DSRCT-18	1	NCT02607813
DSRCT-20	1	NCT03517956, NCT03102320
DSRCT-21	1	NCT02419417
DSRCT-22	1	NCT03517956
DSRCT-23	1	NCT04503278
DSRCT-24	2	NCT04503278
DSRCT-25	1	NCT04503278
DSRCT-26	1	NCT03127215
DSRCT-28	1	NCT04503278
DSRCT-29	1	NCT04503278
DSRCT-30	1	NCT04551521

Supplementary Table 3. SNVs and resulting treatment recommendations

Patient	Gene	Mutation	Allele frequency	Inhibitor class (inhibitor)
DSRCT-01	*FLT1*	c.427C>T, p.P142S	0.07	Multikinase (pazopanib)
DSRCT-01	*EPHA3*	c.1339C>G, p.R447G	0.31	Multikinase (dasatinib)
DSRCT-05	*FGFR4*	c.1907G>T, p.G636V	0.17	Multikinase (pazopanib)
DSRCT-09	*MTOR*	c.6644C>A, p.S2215Y	0.40	mTORC1 (everolimus)

Supplementary Table 4. SBS signatures and resulting treatment recommendations

Patient	Molecular tumor board	Signature[A]	Inhibitor class	Additional biomarker(s)
DSRCT-10	3	SBS3	PARP	None
DSRCT-13	1<br>2	SBS3<br>SBS3	PARP<br>PARP[B]	None<br>None
DSRCT-13	3	SBS3	PARP[B]	SNV in *CDK12*
DSRCT-14	1	SBS8	PARP	Increased *SLFN11* expression
DSRCT-17	1	SBS3<br>SBS8	PARP[B]	Gain and increased expression of *PARP1*, increased *PARP2* expression, deletions of *ATM* and *PALB2*
DSRCT-19	1	SBS2	Immune checkpoint	None
DSRCT-21	1	SBS3	PARP	Deletions of *FANCA*, *BAP1*, *WDR48*, and *TOP2B*
DSRCT-26	1	SBS3	PARP[B]	None
DSRCT-27	1	SBS3<br>SBS8	PARP	Increased *SLFN11* expression

A: Designation according to Human Cancer Signatures v3.4 (https://cancer.sanger.ac.uk/signatures).
B: As part of the TOP-ART trial NCT03127215.

Supplementary Table 5. CNVs and resulting treatment recommendations. TCN, total copy number.

Patient	Gene(s)	Expression	TCN	Ploidy	Alteration	Inhibitor class (inhibitor)
DSRCT-03	*PALB2*<br>*ERCC4*	Absent<br>Absent	3.5<br>3.5	6	Deletion<br>Deletion	PARP
DSRCT-07	*PDGFRB*	Unknown	3	2	Duplication	Multikinase (imatinib)
DSRCT-07	*RICTOR*	Unknown	3	2	Duplication	AKT/mTOR
DSRCT-07	*ERCC1*	Unknown	1	2	Deletion	PARP
DSRCT-13	*MET*	Absent	3	2	Gain	MET (cabozantinib)
DSRCT-16	*BRCA1*<br>*FANCE*<br>*RAD51*	Low<br>Low<br>Low	1.7<br>1.8<br>1.3	2	Deletion<br>Deletion<br>Deletion	PARP
DSRCT-17	*CCND1*	High	3	2	Gain	CDK4/6
DSRCT-18	*PTEN*	Low	2	4	Deletion	mTORC1
DSRCT-25	*PTEN*	Low	3	4	Deletion	mTORC1

Supplementary Table 6. Cancer predisposition genes evaluated for rare germline alterations

AIP	*CREBBP*	*FANCF*	*MUTYH*	*RAD51D*	*SOS1*
AKT1	*CYLD*	*FANCG*	*NBN*	*RB1*	*SPINK1*
ALK	*DDB2*	*FANCI*	*NF1*	*RECQL4*	*STAT3*
ANKRD26	*DDX41*	*FANCL*	*NF2*	*RET*	*STK11*
APC	*DICER1*	*FAS*	*NRAS*	*RHBDF2*	*SUFU*
ATM	*DIS3L2*	*FH*	*NSD1*	*RMRP*	*TERC*
BAP1	*DKC1*	*FLCN*	*NTHL1*	*RPS19*	*TERT*
BARD1	*DOCK8*	*GATA2*	*PALB2*	*RPS24*	*TINF2*
BLM	*EGFR*	*GJB2*	*PARN*	*RPS26*	*TMEM127*
BMPR1A	*ELANE*	*GPC3*	*PDGFRA*	*RTEL1*	*TP53*
BRCA1	*EPCAM*	*HFE*	*PHOX2B*	*RUNX1*	*TRIM37*
BRCA2	*ERCC2*	*HMBS*	*PIK3CA*	*SAMD9*	*TSC1*
BRIP1	*ERCC3*	*HPS1*	*PIK3R1*	*SBDS*	*TSC2*
BUB1B	*ERCC4*	*HRAS*	*PMS2*	*SDHA*	*VHL*
CBL	*ERCC5*	*IDH1*	*POLD1*	*SDHAF2*	*WAS*
CDC73	*ETV6*	*KIT*	*POLE*	*SDHB*	*WRAP53*
CDH1	*EXT1*	*KRAS*	*POLH*	*SDHC*	*WRN*
CDK4	*EXT2*	*MAX*	*POT1*	*SDHD*	*WT1*
CDKN1B	*EZH2*	*MEN1*	*PRKAR1A*	*SETBP1*	*XPA*
CDKN1C	*FANCA*	*MET*	*PRSS1*	*SLX4*	*XPC*
CDKN2A	*FANCB*	*MITF*	*PTCH1*	*SMAD4*	*XRCC2*
CEBPA	*FANCC*	*MLH1*	*PTEN*	*SMARCA4*	
CHEK2	*FANCD2*	*MSH2*	*PTPN11*	*SMARCB1*	
COL7A1	*FANCE*	*MSH6*	*RAD51C*	*SMARCE1*	

Supplementary Table 7. Treatment history of patient DSRCT-28

Line	Regimen	Start	Duration (months)	Best response
1	Cisplatin, 5-fluorouracil, docetaxel	05/2018	4	PR
2	Capecitabine	09/2018	5	NA
3	Nivolumab	04/2019	5	SD
4	Doxorubicin	09/2019	2	PD
5	Nab-paclitaxel, carboplatin	11/2019	11	PR
6	Nivolumab, ipilimumab	10/2020	1	PD
7	Palbociclib, letrozole	01/2021	8	NA
8	Gemcitabine	09/2021	3	PD
9	Capecitabine	11/2021	4	PD
10	Paclitaxel	03/2022	2	PD
11	Pazopanib	06/2022	4	PD
12	VIDE (without doxorubicin)	10/2022	5	SD
13	T-DXd	04/2023	18	PR
14	T-DM1	11/2024	3	NA

NA, not available; PR, partial response; SD, stable disease; PD, progressive disease; T-DM1, trastuzumab emtansin.

Supplementary Table 8. Response to T-DXd in patient DSRCT-28 as determined by ^{18}F-FDG-PET/CT

Date of assessment	Response
04/2023	PR
07/2023	PR
10/2023	PR
01/2024	PR
04/2024	PR
07/2024	PR
10/2024	PD

PR, partial response; PD, progressive disease.

Supplementary Table 9. Treatment history of patient DSRCT-30

Line	Regimen	Start	Duration (months)	Best response
1	VAIA	07/2020	2	PR
2	VIDE	08/2020	4	PR
3	Trabectedin	11/2020	9	PR
4	Topotecan, cyclophosphamide	09/2022	9	PD
5	Irinotecan, temozolomide[A]	01/2023	9	PR
6	T-DXd	08/2023	> 24	PR

A: Until 05/2023.
PR, partial response; SD, stable disease; PD, progressive disease.

Supplementary Table 10. Response to T-DXd in patient DSRCT-30 as determined by ^{18}F-FDG-PET/CT

Date of assessment	Response
11/2023	PR
01/2024	PR
04/2024	SD
07/2024	SD
10/2024	PR
02/2025	SD
05/2025	PD
07/2025	PR

PR, partial response; SD, stable disease; PD, progressive disease.
