# Supplementary material for: Multi-layered molecular profiling informs the diagnosis and targeted therapy of desmoplastic small round cell tumor
Source: Nat Commun. 2026 Apr 9;17:3397. doi: 10.1038/s41467-026-71636-0 (PMC13066396; doi:10.1038/s41467-026-71636-0)
Supplement: Supplementary file 2 — Description of Additional Supplementary Files [file 41467_2026_71636_MOESM2_ESM.pdf]

Description of Additional Supplementary Files

Supplementary Dataset 1: Prior treatment lines of DSRCT patients before enrollment in MASTER

Supplementary Dataset 2: Molecular biomarkers identified through multiomics profiling and corresponding treatment recommendations, including intervention baskets and levels of evidence assigned according to the framework proposed by Leichsenring et al. (2019), as determined by the interinstitutional MTB of the MASTER program
